# Short-term and long-term epidemiological impacts of sustained vector control in various dengue endemic settings: A modelling study

**DOI:** 10.1371/journal.pcbi.1009979

**Published:** 2022-04-01

**Authors:** Haoyang Sun, Joel Koo, Borame L. Dickens, Hannah E. Clapham, Alex R. Cook

**Affiliations:** Saw Swee Hock School of Public Health, National University of Singapore, Singapore, Republic of Singapore; University of Notre Dame, UNITED STATES

## Abstract

As the most widespread viral infection transmitted by the *Aedes* mosquitoes, dengue has been estimated to cause 51 million febrile disease cases globally each year. Although sustained vector control remains key to reducing the burden of dengue, current understanding of the key factors that explain the observed variation in the short- and long-term vector control effectiveness across different transmission settings remains limited. We used a detailed individual-based model to simulate dengue transmission with and without sustained vector control over a 30-year time frame, under different transmission scenarios. Vector control effectiveness was derived for different time windows within the 30-year intervention period. We then used the extreme gradient boosting algorithm to predict the effectiveness of vector control given the simulation parameters, and the resulting machine learning model was interpreted using Shapley Additive Explanations. According to our simulation outputs, dengue transmission would be nearly eliminated during the early stage of sustained and intensive vector control, but over time incidence would gradually bounce back to the pre-intervention level unless the intervention is implemented at a very high level of intensity. The time point at which intervention ceases to be effective is strongly influenced not only by the intensity of vector control, but also by the pre-intervention transmission intensity and the individual-level heterogeneity in biting risk. Moreover, the impact of many transmission model parameters on the intervention effectiveness is shown to be modified by the intensity of vector control, as well as to vary over time. Our study has identified some of the critical drivers for the difference in the time-varying effectiveness of sustained vector control across different dengue endemic settings, and the insights obtained will be useful to inform future model-based studies that seek to predict the impact of dengue vector control in their local contexts.

## Author summary

Sustained vector control remains key to reducing the global burden of dengue. However, current understanding of the main drivers for the differences in the time-varying epidemiological impact of dengue vector control across different transmission settings remains limited. We developed an agent-based model and showed that in the absence of a highly effective intervention technology that is able to eliminate dengue transmission even in an entirely susceptible population, a fixed level of reduction in the *Aedes* abundance would only cause temporary reduction in dengue incidence. Furthermore, the time point at which intervention ceases to be effective is strongly influenced not only by the intensity of vector control and the pre-intervention transmission intensity, but also by the individual-level heterogeneity in biting risk. Besides, the intensity of vector control interacts with the other two factors mentioned earlier, and the interaction magnitude also changes over time. These insights will be useful to inform future modelling studies that seek to predict the impact of *Aedes* control on dengue transmission in their local contexts, and have important implications for the design of intervention strategies to achieve sustained reduction in the global burden of dengue.

## 1. Introduction

As the most widespread viral infection transmitted by the *Aedes* mosquitoes, dengue has been estimated to cause 51 million febrile disease cases globally each year, of whom 3.6 million may require hospitalization [1]. Unfortunately, the only licensed dengue vaccine to date, CYD-TDV, has been found to enhance the future risk of severe dengue infections for seronegative recipients and hence cannot be used to provide protection for all individuals [2]. Even under the very optimistic assumption that 100% of the CYD-TDV vaccine recipients are seropositive, sustained vector control remains key to reducing the burden of dengue [3]. Recently, there has been a growing interest in applying novel vector control technologies to reduce dengue transmission. For instance, combining incompatible and sterile insect techniques has been demonstrated to dramatically reduce the *Aedes* population size by over 90% under field conditions [4–6], and the application of attractive toxic sugar baits has also shown impressive results in both laboratory and small-scale field experiments [7, 8]. Other examples include introducing the wMel *Wolbachia* strain to the *Aedes* population with the aim of reducing vector competence instead of suppressing the mosquito population [9].

Although the novel technologies described above have the potential to achieve dengue elimination even in high-transmission settings, large-scale implementation of these interventions remains challenging. Thus, while more work is still required to evaluate the effectiveness of the novel *Aedes* control methods mentioned earlier, it is also important to assess the short- and long-term impacts of the current vector control measures before more powerful vector control tools become available to be rolled out on a large scale. At present, evidence supporting the epidemiological impact of the existing dengue vector control measures remains limited, due to the lack of surveillance data and the challenge in deploying rigorous study designs to assess intervention effectiveness, as well as insufficient investment in sustained mosquito suppression [10]. In particular, assessing the long-term effectiveness of dengue vector control can be extremely challenging, as this would require substantial investment in monitoring and evaluation. Moreover, long-term dengue vector control trials may violate the principle of equipoise once an intervention under evaluation has been shown to reduce disease incidence in the short to medium term, even though there may be genuine uncertainty regarding its epidemiological impact in the long run.

Although not a replacement for surveillance data, mathematical modelling can serve as a useful tool to compare the predicted dengue incidence with and without interventions, and thus to minimize the confounding bias commonly encountered in studies where high cost and research ethics prohibit the inclusion of a control group for long-term evaluation. So far, very few studies have been carried out to model the impact of dengue vector control in both the short and long terms [10, 11], and to the best of our knowledge, none has examined how the time-varying impact of dengue vector control changes across different transmission settings. Narrowing this knowledge gap will yield useful insights into the important details of a transmission context that must be accounted for to predict the short- and long-term epidemiological impacts of a sustained dengue vector control intervention.

To generate important insights to guide the implementation of both the existing and novel dengue vector control measures, in this study we aim to use mathematical modelling to (i) simulate the change in dengue incidence within a 30-year time period following a sustained reduction in the *Aedes* population size by 10%—90%, and (ii) identify key factors that explain the variation in the short- and long-term vector control effectiveness across different dengue endemic settings.

## 2. Methods

### 2.1 Individual-based model

#### 2.1.1 Overview

**Fig 1:**
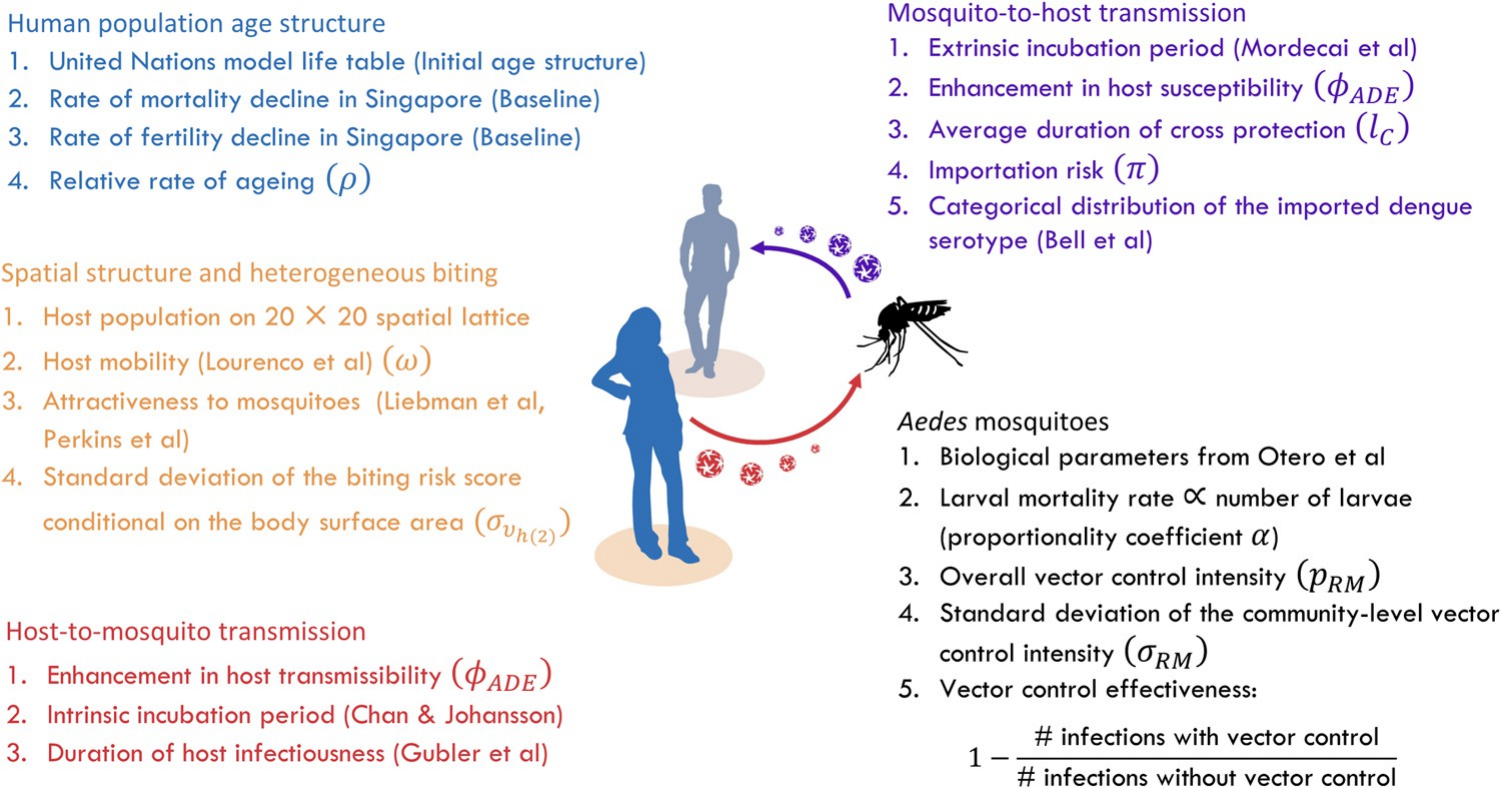
Schematic overview of the individual-based model.

We first simulated the introduction and autochthonous transmission of dengue virus in the absence of vector control for 100 years (“warm-up period”) to establish the baseline immunity in the population (S1 and S2 Figs show the change in dengue incidence and seroprevalence during the 100-year warm-up period under different levels of dengue transmission intensity). Thereafter, we continued the simulation for another 30 years (“intervention period”) with vector control being implemented to maintain the vector density at a reduced level. The discrete time step used to simulate dengue transmission was equal to the length of the mosquito feeding cycle (*l*_*f*_), during which a mosquito took a blood meal, looked for breeding sites, laid eggs, and then searched for the next blood meal [12]. We created different scenarios in our simulations by varying the overall level of vector control intensity, pre-intervention mosquito density, dengue importation rate, rate of change in the population age structure, host mobility rate, and the degree of individual-level heterogeneity in the risk of being bitten by the *Aedes* mosquitoes. In addition, we also varied the assumptions regarding the immunological interactions between dengue serotypes, as well as the degree of spatial heterogeneity in vector control intensity across different simulation runs to assess their influence on the model output (Fig 1).

#### 2.1.2 Dengue infections in humans

Each human was allowed to have at most four sequential infections, with life-long protection against homotypic reinfection and temporary cross-protection against heterotypic infections. Upon receiving an infectious bite, each human had a probability $\tau_{M\to H}^{\left(k\right)}$ of becoming infected in the absence of immunity. Here, the vector-to-host transmission probability depended on the number of historical dengue infections an individual had previously experienced (*k* = 0,1,2,3), where we set $\tau_{M\to H}^{\left(k\right)}$ to be 0.5 for *k*≠1 (i.e., assumed to be equal for primary, tertiary, and quaternary infections for simplicity, due to the challenges of distinguishing between these values using epidemiological data), and 0.5∙(1+*ϕ*_*ADE*_) for *k* = 1 to account for the enhanced susceptibility to secondary infections. On average, each infected human took *l*_*LP*_ days to become infectious and another *l*_*R*_ days to achieve viral clearance, followed by a cross-protection period lasting an average of *l*_*C*_ days (or lifelong immunity against all serotypes after recovery from a quaternary infection); we assumed the length of each of these time intervals to be exponentially distributed.

Similar to the model implemented by Hladish *et al*. to simulate dengue introductions [13], each human had a time-invariant probability *π* of being exposed to dengue virus from an external source in each feeding cycle, and the exposure may be resisted by immunity. To simulate the serotype of the imported dengue virus for each exposed individual, we obtained a random draw from a categorical distribution with the probability parameters equal to the relative frequencies of the four dengue serotypes in Southeast Asia estimated by Bell *et al*. [14]. Here, we assumed the parameters of this categorical distribution to be fixed for the warm-up period, but time-varying for the intervention period. Specifically, we first averaged Bell *et al*.’s estimates across the 45-year study period to be used for the simulation of dengue introductions throughout the 100-year warm-up period [14], since the length of the warm-up period far exceeded the length of the time period for which data were available. Next, we used linear interpolation to transform Bell *et al*.’s quarterly estimates for the period of 1970–2000 into daily estimates [14]. These estimates were then used to simulate the introduced dengue serotypes at each feeding cycle during the 30 years of the intervention period.

#### 2.1.3 Dengue infections in mosquitoes

Each susceptible mosquito had a probability $\tau_{H\to M}^{\left(k\right)}$ of becoming infected after biting an infectious human who had historically been infected *k* times prior to the current infection. We set $\tau_{H\to M}^{\left(k\right)}$ to be 0.5 for *k*≠1, and 0.5∙(1+*ϕ*_*ADE*_) for *k* = 1 to account for the enhanced transmissibility from humans undergoing secondary infections due to increased viral load [15, 16]; we assumed equal degrees of enhancement in transmissibility and susceptibility. Note that in actuality the transmission probabilities between humans and mosquitoes in the absence of enhancement could differ from 0.5, which we used for our individual-based model following previous studies [17, 18]. However, the uncertainty in these values can be absorbed by adjusting the vector-to-host ratio to achieve a certain level of transmission intensity. We assumed the extrinsic incubation period to follow an exponential distribution with mean *l*_*EIP*_(*T*), which was calculated as a deterministic function of temperature (*T*) based on the posterior mean of the parameters estimated for a thermal response model by Mordecai *et al*. [19]. Here, we accounted for the seasonal variation in dengue transmission intensity by allowing the temperature to fluctuate between 25°C and 31°C (i.e., similar to the range of the monthly mean temperature in Bangkok or Phnom Penh) within each year using a sine function. We did not explore alternative temperature ranges in this study, since temperature amplitudes were presumed to be reasonably small in most settings with endemic dengue transmission, and any changes in the transmission intensity due to changes in the mean temperature can be reproduced by adjusting the average vector-to-host ratio. Finally, once a mosquito had survived beyond the extrinsic incubation period of the virus, she was assumed to remain infectious until death.

#### 2.1.4 Spatial structure and heterogeneous biting

We created a set of communities forming on a squared lattice of size 20×20, following the spatial structure specified by Lourenço & Recker [17]. At the start of the simulation, a total of *N*_*H*_ fully susceptible humans and *N*_*BS*_ mosquito breeding sites were uniformly distributed over space (We also conducted a sensitivity analysis to examine the impact of non-uniform distribution of mosquito breeding sites prior to the roll-out of the vector control intervention on the model output; S1 Text has full details). Within each feeding cycle *t*, each adult female mosquito *m* randomly bit a human *h* with probability $p_{Bite}^{t}(m,h)$, which was calculated as follows:

$$p_{Bite}^{t}(m,h)=\left\{ \begin{array}{cc} (1-\omega )∙\frac{\upsilon_{h}^{t}}{\sum_{h^{\prime }}\upsilon_{h\prime }^{t}∙\delta (C[m],C[h^{\prime }])}, & \mathrm{if}\;\delta (C[m],C[h])=1;\\ \omega ∙\frac{\upsilon_{h}^{t}}{\sum_{h^{\prime }}\upsilon_{h\prime }^{t}∙(1-\delta (C[m],C[h^{\prime }]))}, & \mathrm{if}\;\delta (C[m],C[h])=0. \end{array}\right.$$

In the equation above, we used *C*[*h*] to denote the community where human *h* lived (similarly for *C*[*m*]). The value of the indicator function *δ*(*C*[*m*], *C*[*h*]) was defined to be one if the two communities *C*[*m*] and *C*[*h*] were identical or neighbors to each other, and zero otherwise. Given the limited flight range of *Aedes aegypti*, we followed Lourenço & Recker and assumed each mosquito *m* was only able to bite humans present in her own community *C*[*m*] or the neighboring communities [17]. Long-distance transmission occurred due to human movement, and we allowed each mosquito to randomly bite a human living in a distant community with probability *ω* [17]. The numerator $\upsilon_{h}^{t}$ denoted the “biting risk score” of individual *h* at time *t*, which was normalized according to the equation above to obtain $p_{Bite}^{t}(m,h)$. We computed the biting risk score as the product of the following two variables:

$$\upsilon_{h}^{t}=\upsilon_{h(1)}^{t}∙\upsilon_{h(2)}.$$

The first variable $\upsilon_{h(1)}^{t}$ denoted the relative attractiveness to mosquitoes, which was estimated as a function of an individual’s body surface area according to results from Liebman *et al*.’s field study [20], with the growth in body surface area from birth to adulthood simulated based on the age and sex of that individual following Perkins *et al*. [21]. The second variable *υ*_*h*(2)_ accounted for the residual variation in the biting risk score due to other factors, such as differential duration each person spent near biting mosquitoes, wearing of protective clothing, and the individual-level variation in sweat composition and production [22]. For simplicity, we assumed *υ*_*h*(2)_ to be independent of age and generated its value from a gamma distribution with mean one and standard deviation $\sigma_{\upsilon_{h(2)}}$, and we varied the value of $\sigma_{\upsilon_{h(2)}}$ across different transmission settings to study its impact on the short- and long-term effectiveness of vector control (refer to section 2.1.7 for more details).

#### 2.1.5 Human age structure

We simulated the birth, ageing, and death of humans at a time step of 28 days. The maximum human lifespan was set to be 100 years, and each human’s age was treated as an integer variable measured in the unit of 28 days, so that every time step we simulated the demographic changes in the population, each human either had their age increased by one or was removed from the population due to death.

We used the United Nations model life table (General Pattern with life expectancy at birth being 75 years) [23] and assumed a constant mortality rate within each 5-year age interval in the life table. This allowed us to convert the age interval length of the life table to 28 days. Prior to the start of the warm-up period, we initialized the age of each human such that the proportion of individuals falling within each 28-day age interval tallied with the stationary age distribution informed by the life table. Throughout the warm-up period, the number of incident deaths occurring at each age every 28 days was fixed at the corresponding expected value according to the life table, so that the stationary age structure can be maintained. Each death was replaced by a newborn whose sex was randomly generated assuming equal probabilities of male and female births.

For the 30 years of the intervention period, we modelled different rates of changes in the population age structure to examine the associated impact on the vector control effectiveness. Let the annual mortality rate at age *a* in year *y* be *λ*_*a*,*y*_, with *y* = 0 referring to the first year of the intervention period. We assumed *λ*_*a*,*y*_ to decline over time according to the following equation:

$$\log\left(\lambda_{a,y}\right)=\log\left(\lambda_{a,0}\right)+\rho ∙\beta_{a}∙y\;(\rho \geq 0,\;\beta_{a}<0).$$

In the equation above, the constant *β*_*a*_ was defined such that $\left(1-e^{\beta_{a}}\right)$ was equal to the average annual percentage reduction in mortality rate at age *a* in Singapore across the period of 1990–2020 [24]. Hence, the rate of mortality decline can be varied by changing the value of the relative rate of ageing *ρ*, with *ρ* = 1 indicating that the mortality rate declined at the same speed as observed in Singapore. Once we calculated *λ*_*a*,*y*_ based on the values of *a*, *y* and *ρ*, the age-specific probability of death within a 28-day time interval can be derived to simulate mortality events using a binomial distribution.

The same method was applied to create different rates of fertility decline, where we used the same relative rate of ageing *ρ* described earlier. Note that previously we did not specify the age-specific fertility rates since each death was instantaneously replaced by a newborn during the warm-up period. Here, we derived the baseline age-specific fertility rates (i.e., when *y* was equal to zero) such that both of the following conditions were satisfied: (1) the ratios of the baseline fertility rates between different ages tallied with the corresponding ratios observed in Singapore in 1990 [24]; (2) the expected total number of newborns in each 28-day time interval, calculated under the baseline age-specific fertility rates and the stationary age distribution mentioned earlier, tallied with the total number of newborns every 28 days during the warm-up period.

#### 2.1.6 Development of *Ae*. *aegypti* mosquitoes

We modelled the development of *Ae*. *aegypti* mosquitoes using a Markov process with four states: eggs, larvae, pupae, and adults. Within each feeding cycle, each adult female mosquito *m* randomly chose a breeding site located in either her own community *C*[*m*] (i.e., the community where she was born) or one of the neighboring communities to lay eggs. The number of eggs laid in one oviposition followed a Poisson distribution with mean *θ*_*E*_. Male and female adult emergence was assumed to be equally likely, and male mosquitoes were not explicitly modelled upon adult emergence.

The relationship between the mosquito developmental rates and temperature was specified using Schoolfield *et al*.’s model [25]. Following Otero *et al*., the mortality rates of eggs and adult female mosquitoes were both treated as constant, and the larval mortality rate was decomposed into the sum of the natural mortality rate under optimal conditions and the density-dependent mortality rate [26]. We modelled the natural larval mortality rate using an exponential decay function of temperature [26] and assumed the density-dependent larval mortality rate to be proportional to the number of larvae present in a breeding habitat; we denoted the proportionality coefficient by *α*. The pupal mortality rate was also assumed to vary with temperature according to an exponential decay function, with an additional “emergence factor” to account for unsuccessful emergence of adult mosquitoes [26]. The discrete time step used to simulate the development of mosquitoes during the aquatic stage was taken to be one tenth of the mosquito feeding cycle length, to keep the probabilities of multiple transitions (i.e., from eggs to pupae, from eggs to adults, or from larvae to adults) low (<0.05).

At the start of the simulation, the number of eggs in each breeding site was randomly drawn from a Poisson distribution with mean *n*_*E*_, with no larvae, pupae, or adults present. To vary the pre-intervention vector-to-host ratio across different simulation runs, we varied the parameter *α* which was used to calculate the density-dependent larval mortality rate as described earlier. Here, we chose to vary the productivity instead of the total number of breeding sites for the sake of computational efficiency, since the simulation run-time increased with the number of breeding sites. Finally, at the end of the 100-year warm-up period, we randomly selected a proportion (*ξ*_*C*_) of the breeding sites in each community *C* to be permanently removed; the level of vector control intensity *ξ*_*C*_ for each community *C* was independently drawn from a beta distribution with mean *p*_*RM*_ (“overall level of vector control intensity”) and standard deviation *σ*_*RM*_. Although here we implemented vector control via source reduction, the same effect can be achieved using other strategies such as the incompatible insect technique or the sterile insect technique used in real-world settings.

It should be noted that throughout this study, we treated *Ae*. *aegypti* as the sole vector for dengue transmission. In reality, however, *Ae*. *albopictus* may also play an important role in spreading the disease in some endemic areas. While dengue transmission by *Ae*. *albopictus* peaks at a relatively lower temperature compared with *Ae*. *aegypti* [19] and the flight distance of *Ae*. *albopictus* is also relatively longer [27], we chose to focus exclusively on *Ae*. *aegypti* since it is the primary vector for dengue transmission in most endemic settings, and the general conclusions of our study are also unlikely to be substantially affected by the aforementioned differences between the two mosquito species.

#### 2.1.7 Simulation model parameters

We used Latin hypercube sampling [28] to create 1,000 combinations of the following parameters: *π*, *ω*, *ρ*, $\sigma_{\upsilon_{h(2)}}$, *α*, *ϕ*_*ADE*_, *l*_*C*_, and *σ*_*RM*_ to (1) study the impact of dengue importation, human mobility, population ageing, individual-level heterogeneity in the risk of being bitten, and pre-intervention mosquito abundance on vector control effectiveness, and (2) test the sensitivity of our model output with respect to changes in the degree of antibody-dependent enhancement, average duration of cross-protection, and the degree of spatial variability in vector control intensity assumed in the simulation model. For each combination of the eight parameters mentioned earlier, dengue transmission during the warm-up period was simulated only once. Thereafter, we simulated dengue transmission during the intervention period ten times, each time with a different overall level of vector control intensity quantified by *p*_*RM*_. The set of values taken by *p*_*RM*_ contained zero, which represented the counterfactual scenario to be used for the calculation of vector control effectiveness as described in the next section (Refer to Table 1 for the list of parameter values). The simulation model was implemented using Python 3.9 (S1 Code), and the average time for a simulation run during the warm-up period and the intervention period was 14,667s (4h 4mins) and 4,400s (1h 13mins) respectively. Simulations were carried out in parallel across 16 compute nodes on a High-Performance Computing cluster, with each node consisting of 24 Intel(R) Xeon(R) CPU E5-2680 v3 @ 2.50Ghz single-thread processors.

### 2.2 Analyses of the simulation output

To measure the overall dengue transmission intensity prior to the implementation of vector control in each simulation run, we computed the proportion of nine-year olds who had prior dengue exposure at the end of the warm-up period (“baseline PE9”). We also calculated the increase in the median age of the entire population during the 30-year intervention period (Δ_age_). The baseline PE9, Δ_age_, and the simulation model parameters *p*_*RM*_, *π*, *ω*, $\sigma_{\upsilon_{h(2)}}$, *ϕ*_*ADE*_, *l*_*C*_, and *σ*_*RM*_ (hereafter referred to as the “predictor variables”) were assessed in their contribution to explaining the variability in the vector control effectiveness, as explained later in this section.

We extracted the monthly number of dengue infections from each simulation run and defined the vector control effectiveness (provided *p*_*RM*_>0) within a time window (*t*_*y*_, *t*_*y*_+Δ_*t*_] as follows:

$${eff}_{p_{RM}}(t_{y},\;t_{y}+\Delta_{t}]=1-\frac{\#\;of\;infections\;within(t_{y},\;t_{y}+\Delta_{t}]\;with\;vector\;control}{\#\;of\;infections\;within(t_{y},\;t_{y}+\Delta_{t}]\;without\;vector\;control}.$$

In the equation above, we used $t_{y}\in \left\{0,\frac{1}{12},\frac{2}{12},\ldots ,\frac{358}{12},\frac{359}{12}\right\}$ to denote the number of years between the start of the intervention period and the left endpoint of the time window, and $\Delta_{t}\in \{\frac{1}{12},\frac{2}{12},\ldots \frac{360}{12}\}$ to denote the time window length in years, subject to the constraint *t*_*y*_+Δ_*t*_≤30. For each simulation where *p*_*RM*_>0, we calculated the vector control effectiveness within each of the following time windows: (0, 5], (5, 10], (10, 15], (15, 20], (20, 25], (25, 30], and (0, 30]. In addition, we obtained the “critical time point” $t_{y}^{*}$ defined as the minimum value of *t*_*y*_ such that the intervention effectiveness within the time window (*t*_*y*_, *t*_*y*_+5] was negative, to represent the time point at which the intervention stopped having an impact:

$$t_{y}^{*}=\min\left\{t_{y}:\;t_{y}\in \{0,\frac{1}{12},\frac{2}{12},\ldots ,\frac{299}{12},\frac{300}{12}\}\wedge {eff}_{p_{RM}}\left(t_{y},t_{y}+5\right]<0\right\}.$$

Due to the multi-annual incidence patterns of dengue, we set the length of the time window to be five years in the equation above to reduce noise in the estimated underlying trend in the vector control effectiveness. In the rare event that right censoring occurred (i.e., eff_*p_RM_*_(*t*_*y*_, *t*_*y*_+5]≥0, ∀*t*_*y*_), we set the value of the critical time point to be 25. Hereafter, the intervention effectiveness calculated for each of the seven time windows mentioned earlier, as well as the critical time point, will be referred to as the “outcome variables”, and we used ***y***_***j***_ = (*y*_1*j*_,…,y_9000 *j*_)^*T*^ to denote the vector containing all the values of an outcome variable *j* (*j* = 1,…,8) obtained from the 9,000 simulation runs where vector control was implemented (i.e., *p*_*RM*_>0). Similarly, the predictor variable matrix was denoted by **X** = (*x*_*si*_) (*s* = 1,…,9000, *i* = 1,…,9).

For each overall level of vector control intensity (*p*_*RM*_), we summarized the distribution of each outcome variable using a boxplot. In addition, we also used the extreme gradient boosting (XGBoost) algorithm to capture the nonlinear and interaction effects of the predictor variables on each of the eight outcome variables [29]. As a tree ensemble model, XGBoost is able to capture the complex data dependencies while minimizing over-fitting via regularization [29]. To train the XGBoost model for each outcome variable *j*, we first split our data (**X**, ***y***_***j***_) into training and test sets using a 9:1 ratio and tuned the following hyper-parameters via 5-fold cross validation performed on the training set, where we used the mean absolute error as the evaluation metric: the maximum tree depth, the minimum number of observations in each node, the fractions of rows and columns to be randomly sampled to grow each tree, and the learning rate. Given the optimal hyper-parameters, the number of boosting rounds for the final model was determined by monitoring the out-of-sample prediction accuracy evaluated on the test set.

Finally, to interpret the predictions of the XGBoost model, we calculated the “Shapley additive explanations” (SHAP) value *S*_*ijs*_ for each combination of predictor variable *i*, outcome variable *j*, and simulation run *s* [30]. Unlike partial dependence plot, SHAP value accounted for interaction effects by measuring the impact of each predictor variable on the model prediction for each single sample (in our case, each simulation run) [30]. Specifically, the SHAP value *S*_*ijs*_ quantified the change in the *predicted* value of *y*_*sj*_ due to *x*_*si*_, where the comparator was set to be the predicted value of *y*_*sj*_ had the predictor variable *i* been excluded from the XGBoost model. For each pair of predictor variable *i* (that was not *p*_*RM*_) and outcome variable *j*, we plotted the SHAP values (*S*_*ijs*_) against the values of the predictor variable *i* (*x*_*si*_). Thereafter, to investigate potential interaction between the overall level of vector control intensity (*p*_*RM*_) and each of the other predictor variables, for each value taken by *p*_*RM*_ we fitted a smoothing spline to the corresponding subset of data points in each scatterplot, to visually examine whether the fitted splines under different values of *p*_*RM*_ differed from one another. All the analyses of the simulation output were performed using Python 3.9.

**Table 1:** List of parameter values for the simulation model.

Constant parameters			
Notation	Description	Value	Remark
*N*_*H*_	Total number of humans at initialization	100,000	—
*N*_*BS*_	Total number of breeding sites at initialization	100,000	—
*n*_*E*_	Average number of eggs per breeding site at initialization	500	Approximately equal to the number of eggs per breeding site at the peak breeding season in Otero *et al*.’s simulation model [26]
*l*_*f*_	Length of the mosquito feeding cycle (days)	4	[12] Assumed constant within (25°C, 31°C)
*l*_*LP*_	Average duration of the human latent period (days)	6	Approximated using the intrinsic incubation period [31]
*l*_*R*_	Average duration of human infectiousness (days)	5	[32]
*θ*_*E*_	Average number of eggs laid by an *Aedes* mosquito in one oviposition	63	[26]
*μ*_*E*_	Mortality rate of mosquito eggs (per day)	0.011	[26]
*ef*	Emergence factor (Proportion of pupae reaching maturation that emerged as adults)	0.83	[26]
*μ*_*F*_	Mortality rate of adult female mosquitoes (per day)	0.091	[26]
Parameters whose values were varied across simulations			
Notation	Description	Range	Remark
*π*	Probability of being exposed to dengue virus from an external source per human per cycle	(2^0^,2^4^) × 10^−5^	Drawn from a uniform distribution on a log_2_ scale
*ω*	Probability of biting a human living in a distant community per mosquito per bite	(10^−4^, 10^−1^)	Drawn from a uniform distribution on a log_10_ scale
*ρ*	Relative rate of ageing	Unif (0,4)	Given the same age structure at the beginning of the intervention period, the median age of the human population after 30 years ranged from 38 to 51 years old across the 10,000 simulations
$\sigma_{\upsilon_{h(2)}}$	Standard deviation of the biting risk score controlling for body surface area	Unif (0,1)	The range was restricted to (0,1) to avoid a high degree of collinearity between the baseline PE9 and $\sigma_{\upsilon_{h(2)}}$
*α*	Proportionality coefficient used to compute the density-dependent larval mortality rate	Unif (0.1, 0.4)	—
*ϕ*_*ADE*_	Susceptibility enhancement and transmissibility enhancement (assumed equal)	Unif (0,1)	Sensitivity analysis
*l*_*C*_	Average duration of cross-protection (days)	Unif (1, 365)	Sensitivity analysis
*σ*_*RM*_	Standard deviation of the community-level vector control intensity	Unif (0, 0.2)	Sensitivity analysis
*p*_*RM*_	Overall level of vector control intensity (proportion of breeding sites removed)	(*)	—

(*) For each combination of the eight parameters above, we simulated dengue transmission during the intervention period 10 times, each time with a different value of *p*_*RM*_ from the set {0, 0.1, 0.2, …, 0.8, 0.9}.

## 3. Results

**Fig 2:**
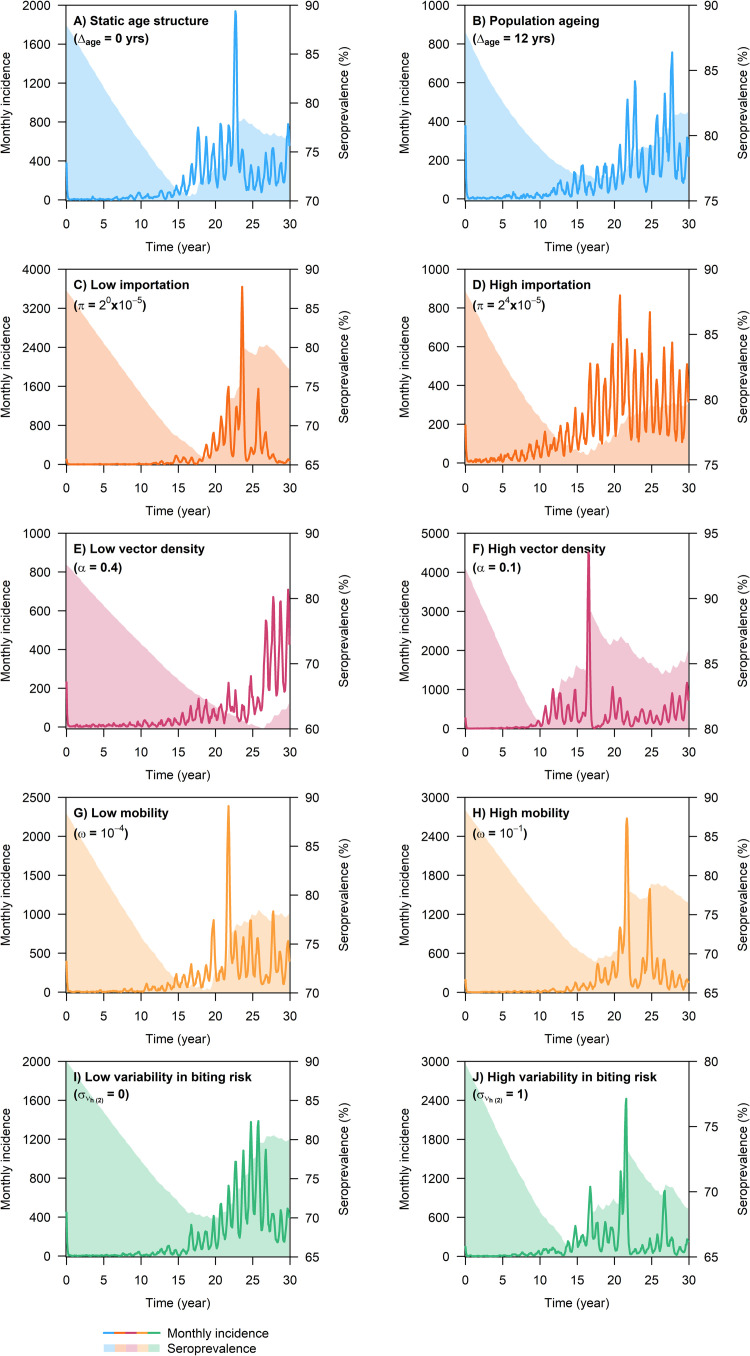
Simulated monthly number of dengue infections and dengue seroprevalence following a permanent reduction in the mosquito population size by 70% in different dengue endemic settings. Unless otherwise stated, model parameters were fixed at the following values: *π* = 4×10^−5^, *ω* = 0.005, *ρ* = 0, $\sigma_{\upsilon_{h(2)}}=0.5,$ *α* = 0.25, *ϕ*_*ADE*_ = 0.5, *l*_*C*_ = 180, *p*_*RM*_ = 0.7, *σ*_*RM*_ = 0 (Here we used *σ*_*RM*_ = 0 to denote spatially uniform vector control intensity).

**Fig 3:**
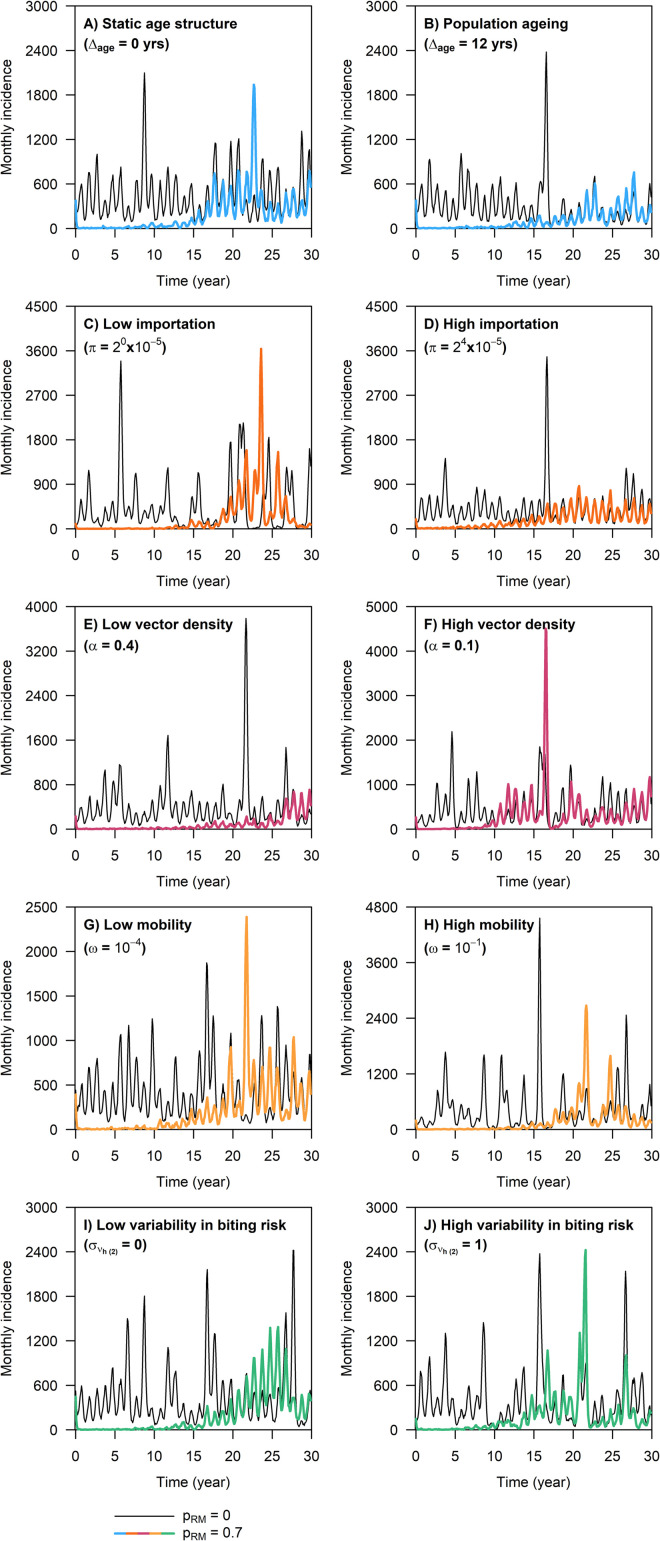
Simulated monthly number of dengue infections during the 30-year intervention period in different dengue endemic settings. Each figure panel showed model outputs obtained from two simulation runs with the same starting condition, where one simulation was performed without vector control and the other with the vector population size permanently reduced by 70% at the start of the intervention period (identical to the corresponding model output shown in Fig 2).

Following a sustained reduction in the mosquito population size, the monthly number of dengue infections immediately dropped to near zero and continued to hover just above zero for a number of years, accompanied by a gradual decline in dengue seroprevalence in the population (Fig 2). In most simulation runs where the overall level of vector control intensity was 0.8 or higher, dengue transmission remained to be suppressed throughout the entire 30-year intervention period (Table 2). By contrast, under a permanent reduction in the mosquito population size by 70% or less (i.e., *p*_*RM*_≤0.7), there was a high probability that dengue incidence bounced back to the pre-intervention level within 30 years (Table 2), with a marked difference in the time point at which vector control ceased to be effective (i.e., critical time point) across different transmission settings (Fig 3 shows the general model behavior).

**Table 2:** Number of simulations where the 5-year intervention effectiveness remained positive throughout the entire 30-year intervention period. For each overall level of vector control intensity, a total of 1000 simulations were performed.

Overall level of vector control intensity (*p*_*RM*_)	0.1	0.2	0.3	0.4	0.5	0.6	0.7	0.8	0.9
Number of simulations where the 5-year intervention effectiveness remained positive throughout the 30-year intervention period	0	0	0	0	1	30	261	704	994

**Fig 4:**
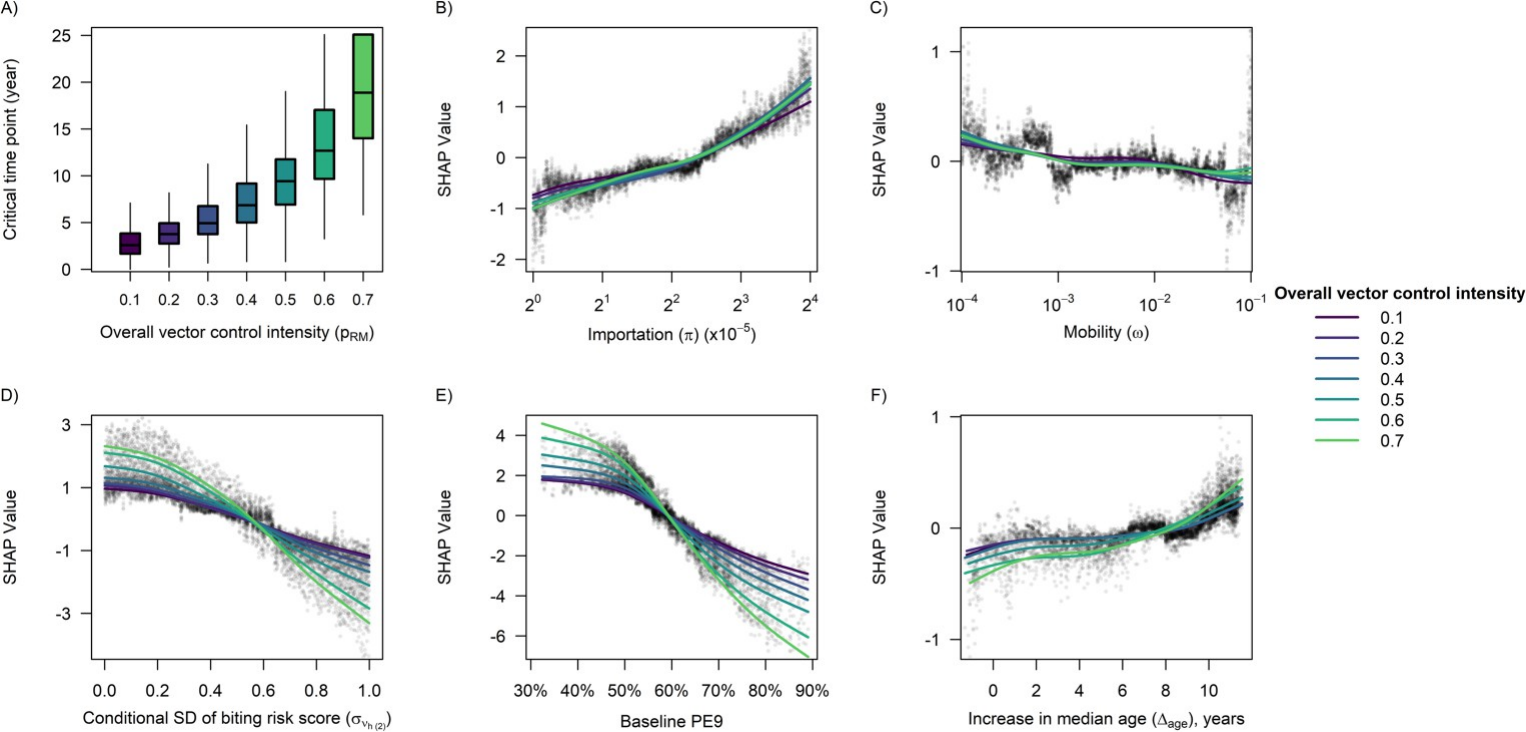
Impact of different predictor variables on the critical time point. For (A), the original values of the critical time point obtained from the simulations were summarized. Each box was drawn from the first quartile (Q1) to the third quartile (Q3) of the data, with the horizontal line in the middle showing the median. The upper whisker boundary was the largest observation that was within Q3 + 1.5 · (Q3—Q1), and the lower whisker boundary was the smallest observation above Q1–1.5 · (Q3—Q1). Data points beyond the whisker boundaries were omitted from the graph for clarity. For (B)—(F), we used SHAP values to quantify the change in the predicted critical time point due to the value of each predictor variable, where each point represents a single simulation run and each curve was obtained by fitting a smoothing spline to the set of data points corresponding to a given overall level of vector control intensity. We excluded simulations where the overall level of vector control intensity was 0.8 or higher, due to the high probability that vector control remained effective throughout the 30-year intervention period.

**Fig 5:**
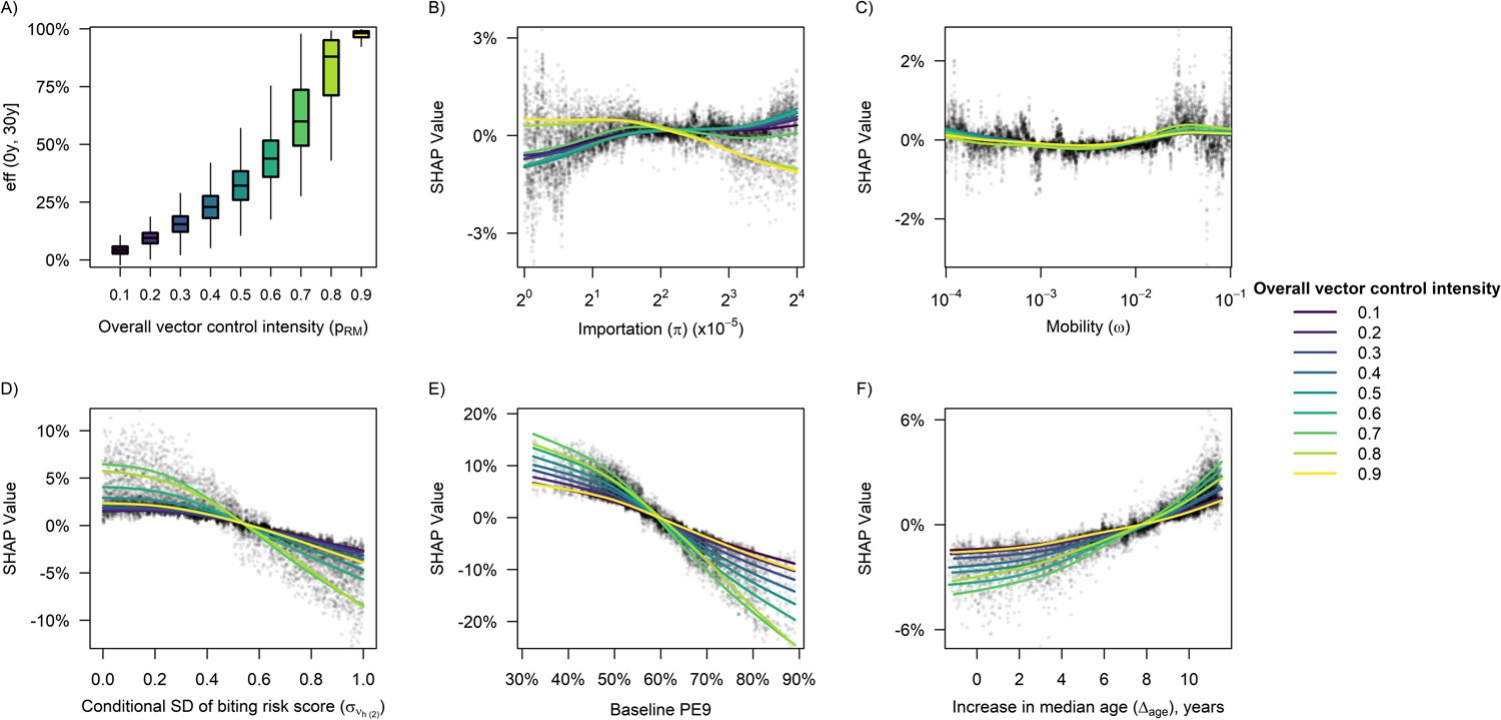
Impact of different predictor variables on the vector control effectiveness across the entire 30-year intervention period. For (A), the original values of the intervention effectiveness obtained from the simulation runs were summarized. Each box was drawn from the first quartile (Q1) to the third quartile (Q3) of the data, with the horizontal line in the middle showing the median. The upper whisker boundary was the largest observation that was within Q3 + 1.5 · (Q3—Q1), and the lower whisker boundary was the smallest observation above Q1–1.5 · (Q3—Q1). Data points beyond the whisker boundaries were omitted from the graph for clarity. For (B)—(F), we used SHAP values to quantify the change in the predicted overall intervention effectiveness due to the value of each predictor variable, where each point represents a single simulation run and each curve was obtained by fitting a smoothing spline to the set of data points corresponding to a given overall level of vector control intensity.

We found the overall level of vector control intensity and the baseline PE9 to be the most important predictors for the critical time point, followed by the degree of individual-level heterogeneity in the biting risk score (Fig 4). It should be noted that in Fig 4, we excluded simulation runs where the overall level of vector control intensity was 0.8 or higher, due to the high probability that vector control remained effective throughout the 30-year intervention period. We also observed that the higher the overall level of vector control intensity (provided *p*_*RM*_≤0.7), the more pronounced the negative impacts of baseline PE9 and heterogeneous biting on the critical time point (Fig 4). The above findings also held true for the case of vector control effectiveness across the entire 30 years of the intervention period (“overall intervention effectiveness”), provided the overall level of vector control intensity was below 0.8 (Fig 5).

**Fig 6:**
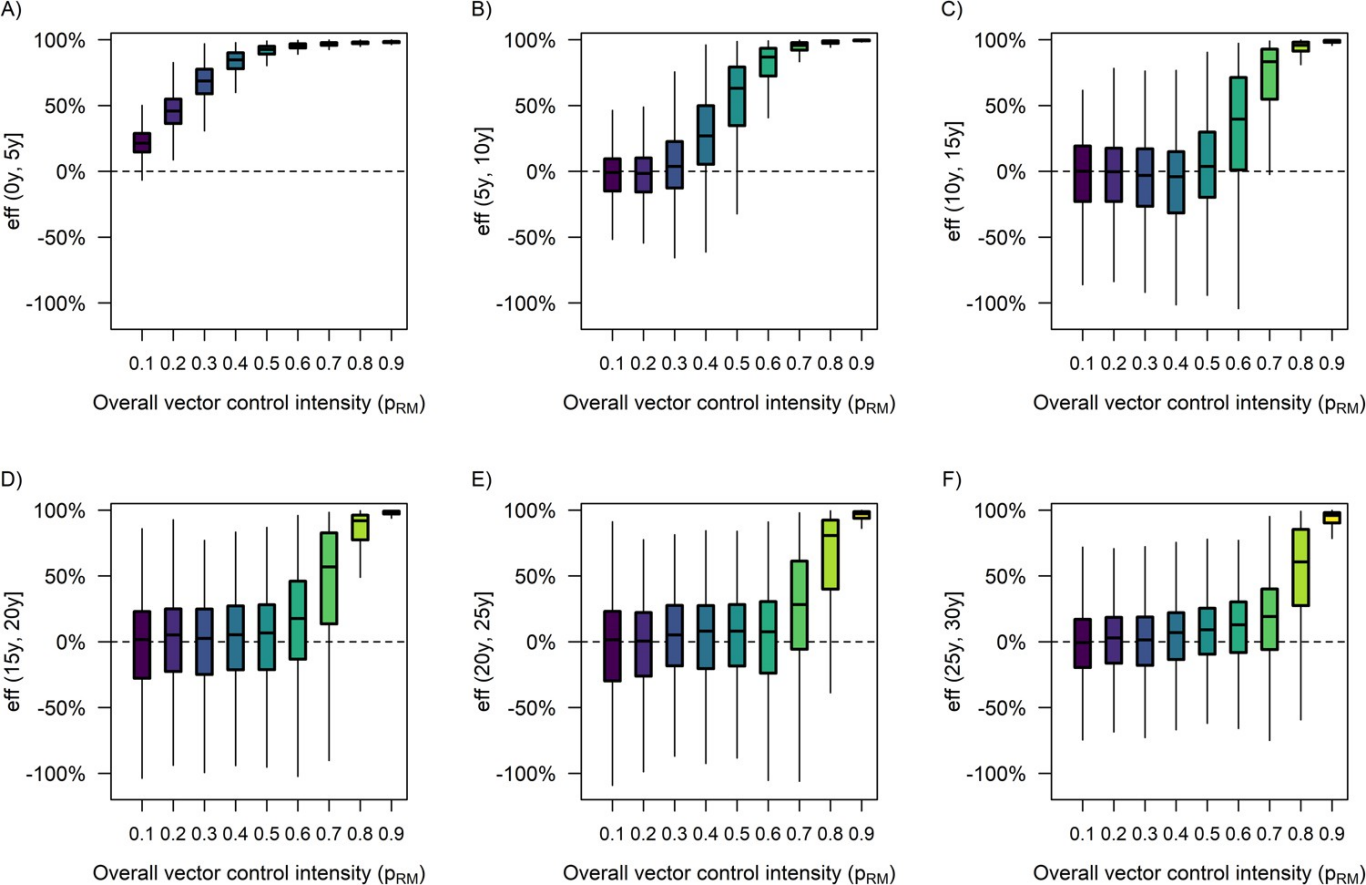
**Distribution of the simulated intervention effectiveness under different overall levels of vector control intensity within each of the following time windows**: (A) (0y, 5y], (B) (5y, 10y], (C) (10y, 15y], (D) (15y, 20y], (E) (20y, 25y], (F) (25y, 30y]. Each box was drawn from the first quartile (Q1) to the third quartile (Q3) of the data, with the horizontal line in the middle showing the median. The upper whisker boundary was the largest observation that was within Q3 + 1.5 · (Q3—Q1), and the lower whisker boundary was the smallest observation above Q1–1.5 · (Q3—Q1). Data points beyond the whisker boundaries were omitted from the graph for clarity.

During the first five years of the intervention period, vector control was substantially more effective when implemented at a higher intensity (Fig 6). However, the median value of intervention effectiveness diminished towards zero over time and eventually became almost indistinguishable between different overall levels of vector control intensity (except for *p*_*RM*_≥0.8) in (25y, 30y] (Fig 6). Interestingly, the median value of intervention effectiveness was found to be negative within the time window (10y, 15y] when the overall level of vector control intensity was equal to 0.3 or 0.4 (Fig 6), which may be due to the low herd immunity in the population that caused dengue incidence to drastically increase, thereby temporarily exceeding the incidence in the counterfactual scenario.

**Fig 7:**
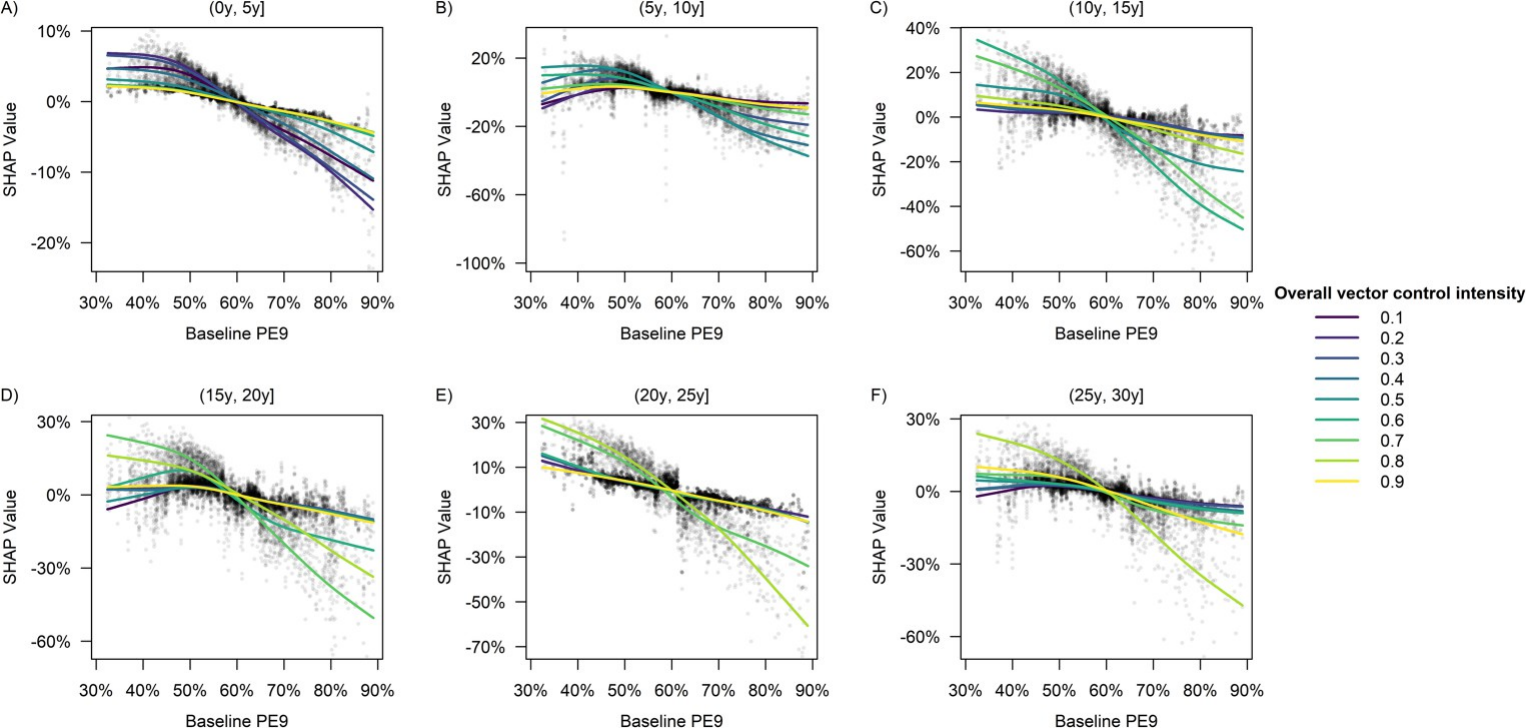
**Impact of baseline PE9 on the predicted vector control effectiveness within each of the following time windows**: (A) (0y, 5y], (B) (5y, 10y], (C) (10y, 15y], (D) (15y, 20y], (E) (20y, 25y], (F) (25y, 30y]. The baseline PE9 referred to the proportion of nine-year olds who had prior dengue exposure at the end of the warm-up period. Each point represents a single simulation run, and the SHAP value quantified the change in the predicted vector control effectiveness due to the value of baseline PE9. Each curve was obtained by fitting a smoothing spline to the set of data points corresponding to a given overall level of vector control intensity.

**Fig 8:**
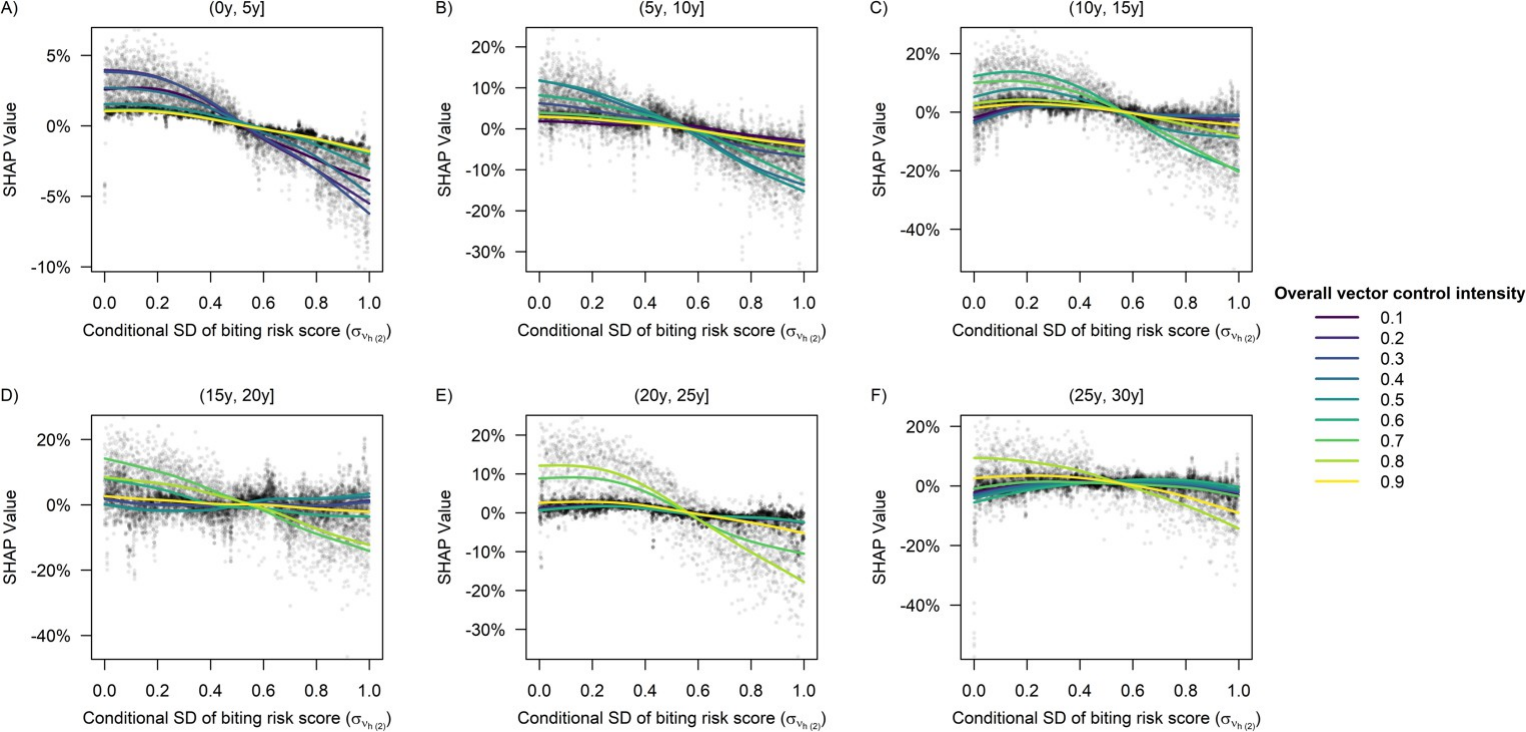
**Impact of individual-level heterogeneity in the biting risk score on the predicted vector control effectiveness within each of the following time windows**: (A) (0y, 5y], (B) (5y, 10y], (C) (10y, 15y], (D) (15y, 20y], (E) (20y, 25y], (F) (25y, 30y]. The parameter $\sigma_{\upsilon_{h(2)}}$ referred to the standard deviation of the biting risk score controlling for body surface area. Each point represents a single simulation run, and the SHAP value quantified the change in the predicted vector control effectiveness due to the value of $\sigma_{\upsilon_{h(2)}}$. Each curve was obtained by fitting a smoothing spline to the set of data points corresponding to a given overall level of vector control intensity.

Within each of the 5-year time windows in Fig 7, we observed an overall inverse relationship between the baseline PE9 and intervention effectiveness, with the impact of baseline PE9 being more pronounced at a low level of vector control intensity (*p*_*RM*_ = 0.2) during (0y, 5y], moderate vector control intensity (*p*_*RM*_ = 0.5) during (5y, 10y], moderately high intensity (*p*_*RM*_ = 0.6 or 0.7) during (10y, 20y], and high intensity (*p*_*RM*_ = 0.8) during (20y, 30y] (Fig 7). Similar conclusions also held true for the relationship between heterogeneous biting and vector control effectiveness (Fig 8). In addition, neither dengue importation risk, nor population ageing, nor host mobility was found to have a substantial impact on vector control effectiveness within any of the 5-year time windows considered in this study (S3–S5 Figs), although a 12-year increase in the median age of the population during the 30-year intervention period was estimated to increase the overall intervention effectiveness by ~8 percentage points on average at a moderately high level of intervention intensity (*p*_*RM*_ = 0.7) (Fig 5).

When we increased the degree of antibody-dependent enhancement in our simulations, there was a modest decrease in both the short-term and the overall intervention effectiveness, as well as the value of the critical time point (S6 and S7 Figs). Furthermore, the average duration of cross-protection was found to have a negligible impact on both the intervention effectiveness and the critical time point (S7 and S8 Figs). Overall, varying the strengths of immune interactions did not result in a substantial change in our model results. Similarly, our intervention effectiveness and critical time point estimates were reasonably robust with respect to changes in the degree of spatial variability in vector control intensity (S7 and S9 Figs) as well as the spatial distribution of mosquito breeding sites (and thus vector-to-host ratios) prior to the roll-out of the vector control intervention (Table A in S1 Text).

## 4. Discussion

Our study highlights the contrast between the short- and long-term impacts of sustained dengue vector control in endemic settings, in congruence with the findings from previous studies: Following the suppression of the *Aedes* mosquito population, the equilibrium between the basic reproduction number and the level of herd-immunity was disrupted, causing dengue incidence to drastically decline [10, 11]. This in turn reduced population immunity over time as new births were added to the susceptible pool and immune individuals were gradually removed from the population due to death [10, 11]. Consequently, in the vast majority of the simulation runs where the mosquito population size was reduced by less than 70%, dengue incidence gradually bounced back and the existing intervention was no longer effective in the end, requiring the intensity of vector control to be further enhanced to reduce dengue transmission [10]. We have additionally demonstrated that the time point at which vector control stopped having an impact depended mainly on the degree of mosquito suppression, pre-intervention transmission intensity, and the degree of individual-level heterogeneity in the risk of being bitten by the *Aedes* mosquitoes. It is thus important for future studies to account for the effects of these variables, as well as their interactions, to forecast the time-varying impact of an *Aedes* control program for planning purposes.

We have shown that a higher pre-intervention transmission intensity was generally associated with a lower proportion of dengue infections averted within each of the 5-year time windows considered in this study. A likely explanation is that an increase in the pre-intervention transmission intensity will generally reduce the average number of years before sustained transmission resumes, thereby causing intervention effectiveness to decrease: Suppose the basic and effective reproduction numbers prior to the intervention are *R*_0_ and 1 respectively (*R*_0_>1), and hence the fraction of the population being susceptible to dengue at baseline is approximately 1/*R*_0_. If vector control reduces the basic reproduction number to *R*_0_/*k* (*R*_0_>*k*>1) and if the susceptible fraction of the population increases by *c* each year during the early phase of the intervention period, the number of years before the effective reproduction number bounces back to one will be $\frac{k-1}{R_{0}∙c}$, which is inversely proportional to *R*_0_. While the above conclusion may rely on some simplifying assumptions, our individual-based model has produced similar results, which showed that the baseline PE9 had a negative relationship with the critical time point especially at a moderately high level of vector control intensity. It should be noted that the relationship between baseline PE9 and the vector control effectiveness was found to be very weak under an extremely high level of vector control intensity (i.e., *p*_*RM*_ = 0.9), which was likely due to the high probability that the intervention was able to reduce the basic reproduction number to below one thereby achieving long-term elimination of dengue, regardless of the baseline PE9 value.

In addition to the important role of pre-intervention transmission intensity in modifying the effectiveness of dengue vector control, our results suggest that the degree of individual-level heterogeneity in the biting risk also needs to be accounted for to accurately forecast the epidemiological impact of a vector control program (unless the intervention is implemented at a very high level of intensity). Earlier studies have produced strong evidence for the heterogeneous blood feeding patterns of the *Aedes* mosquitoes using DNA profiling of bloodmeals [20, 33], and have identified characteristics of individual hosts that may help to improve the accuracy of biting risk predictions (e.g., body surface area, time spent near biting mosquitoes, and the composition of skin microbiota) [20, 22]. Other studies have demonstrated the important implications of heterogeneous biting for vector control: For example, maximum impact will not be achieved if a uniform strategy is implemented in settings where a small number of individuals have a disproportionately large contribution to transmission [34]. Besides, heterogeneous biting may increase the success rate of pathogen invasion, thus making disease eradication more difficult [35]. The results obtained in our study highlight another challenge posed by heterogeneous biting: When the biting risk is more heterogeneous at the individual level, there will be a higher chance for multiple infectious mosquito bites to land on the same susceptible individual, and the resulting infection cannot be averted unless most, if not all, of the aforementioned mosquito bites are prevented. Hence, given a fixed level of reduction in vector density, the percentage decrease in the number of incident dengue infections will be lower in the presence of heterogeneous biting compared with the case where each infectious mosquito chooses to bite a unique individual. Note that to avoid a high degree of collinearity between the baseline PE9 and the variability in biting risk, we set the maximum standard deviation of the biting risk score conditional on the body surface area to be 1, which corresponds to a setting where 55% of the human population receive 90% of all the mosquito bites. In reality, the distribution of mosquito bites may be more skewed in some transmission settings, where the negative impact of heterogeneous biting on the vector control effectiveness can be even larger than what we estimated in this study. More research that quantifies and explains the individual-level heterogeneity in biting risk would be helpful to inform the development of more effective dengue vector control policies that can be implemented to achieve the desired epidemiological impact.

We have observed a weak positive impact of population ageing on the effectiveness of vector control over the 30-year intervention period. However, this should not be interpreted as a minor effect of demographic transition on dengue epidemiology, as previous work has shown that the decline in birth and death rates can cause a substantial decrease in the force of infection, as well as a change in both the age distribution of dengue cases and the periodicity of dengue outbreaks [36]. Instead, our results suggest that population ageing may play a relatively limited role in modifying the effect of a vector control program on dengue incidence. Similarly, we did not find host mobility to have an appreciable impact on vector control effectiveness despite its important influence on the epidemic variability and the risk of serotype extinction [17]. In other words, although both population ageing and host mobility play an important role in influencing dengue transmission, it is likely that the resulting magnitude of change in the level and pattern of dengue incidence does not strongly depend on whether vector control is implemented. Hence, neither of the aforementioned factors was found to substantially modify the intervention impact in our simulations. Furthermore, our study has shown that the average duration of cross-immunity has a minimal impact on vector control effectiveness, while the impact of antibody-dependent enhancement was found to be somewhat stronger. Sensitivity analysis should remain an important component of future modelling studies to assess the uncertainty in the predicted effectiveness of dengue vector control with respect to the uncertainty in the strengths of immune interactions.

In the absence of highly effective vector control tools that are currently available to be rolled out on a large scale to achieve dengue elimination, the results obtained in this study highlight the need to adopt an integrated approach to achieve a sustained reduction in dengue transmission. Special considerations are needed to maximize the cost-effectiveness of combined interventions in each particular context, given that there may be interactions between different intervention strategies. For example, the effectiveness of the CYD-TDV vaccine was predicted to become lower when combined with targeted indoor residual spraying due to the reduced rate of natural infection [37]. Nonetheless, the combination was shown to outperform either alone in high-transmission settings [37], where it may be still beneficial to consider introducing a combined intervention program in view of the relatively low vector control effectiveness based on our model estimates.

Our results need to be interpreted in the light of the following limitations. While we have selected our predictor variables based on the factors that were previously shown to have a potentially important influence on dengue transmission [17, 18, 20, 36, 38], we have also made several simplifying assumptions in our simulations to reduce computational complexity. For example, we modelled the host population structure using a squared lattice of size 20×20, and following Lourenço & Recker, each mosquito was only allowed to bite humans present in her own community or the neighboring communities [17]. Given that the average maximum flight distance of *Ae*. *aegypti* was estimated to be ~333m [27], our lattice was only 2km—3km wide. Although such a spatially constrained area may not have a substantial impact on our model results considering the very weak relationship between host mobility and vector control effectiveness estimated by our model, more work will be needed to ascertain the extent to which enlarging the lattice would change the results. Besides, we assumed temperature to fluctuate between 25°C and 31°C yearly according to a sine function. While the effect of changes in the annual mean temperature alone can be reproduced by adjusting the average vector-to-host ratio, which we varied across simulation runs, changing the intra-annual variation in temperature and accounting for more realistic temperature patterns may cause a modest change in the critical time point estimate provided the baseline PE9 is held fixed. In addition, we also did not consider alternative age structures in the warm-up period of the simulations, and hence the estimated magnitude of interaction between demographic transition and vector control may not generalize to other settings.

Other simplifying assumptions made in our simulations include treating the overall level of vector control intensity as a constant throughout the 30-year intervention period in each simulation run. Scenario planning would need to be incorporated in future work, to predict the change in the incidence of dengue infections due to unforeseen circumstances such as failure to achieve sustained mosquito suppression. We have also used the relative frequencies of the four dengue serotypes in Southeast Asia to simulate dengue importation, and the serotype composition of the imported dengue viruses in other geographical areas can be significantly different from what we assumed in the model. Nonetheless, we believe the main findings obtained in this study have important implications for vector control policy planning in most dengue endemic areas in the world, since the mechanisms underlying (1) the difference between the short-term and long-term impacts of sustained dengue vector control and (2) the influence of baseline transmission intensity and heterogeneous biting on intervention effectiveness, as we had discussed earlier, are unlikely to be dependent on the simplifying assumptions made in our model. Finally, we may have overestimated the variability in the distribution of vector control effectiveness due to the highly stochastic outputs from the simulation model. Hence, our study has mainly focused on the average effect of each predictor variable on the predicted intervention effectiveness, which we derived separately under different levels of vector control intensity.

In conclusion, we used a detailed individual-based model to demonstrate the contrast between the short-term and long-term impacts of sustained vector control in dengue endemic settings. The finding that short-term gains from vector control (unless implemented at a very high level of intensity) are mostly reversed as population immunity wanes may lie behind the resurgence of dengue in settings such as Singapore where previously successful vector control created a generation with very little immunity to dengue. Our model has also identified some of the critical factors that influence the effectiveness of vector control, and how the magnitudes of their impacts may vary over time under different levels of vector control intensity. These insights will be useful to inform future model-based studies that seek to predict the impact of dengue vector control in their local contexts, and have important implications for the design of intervention strategies to achieve sustained reduction in the global burden of dengue.

## Supporting information

S1 Fig Change in dengue incidence during the 100-year warm-up period under different levels of dengue transmission intensity. In all the figure panels, the yearly dengue incidence was found to converge within 20 years.
(TIFF)

S2 Fig Change in dengue seroprevalence during the 100-year warm-up period under different levels of dengue transmission intensity. The four simulation runs were identical to those that generated the dengue incidence shown in S1 Fig, and in all the figure panels, dengue seroprevalence converged within 20 years.
(TIFF)

S3 Fig **Impact of dengue importation on the predicted vector control effectiveness within each of the following time windows**: (A) (0y, 5y], (B) (5y, 10y], (C) (10y, 15y], (D) (15y, 20y], (E) (20y, 25y], (F) (25y, 30y]. The parameter *π* referred to the probability of being exposed to dengue virus from an external source per human per feeding cycle. Each point represents a single simulation run, and the SHAP value quantified the change in the predicted vector control effectiveness due to the value of *π*. Each curve was obtained by fitting a smoothing spline to the set of data points corresponding to a given overall level of vector control intensity.
(TIFF)

S4 Fig **Impact of population ageing on the predicted vector control effectiveness within each of the following time windows:** (A) (0y, 5y], (B) (5y, 10y], (C) (10y, 15y], (D) (15y, 20y], (E) (20y, 25y], (F) (25y, 30y]. The parameter Δ_age_ referred to the increase in the median age of the population during the 30-year intervention period. Each point represents a single simulation run, and the SHAP value quantified the change in the predicted vector control effectiveness due to the value of Δ_age_. Each curve was obtained by fitting a smoothing spline to the set of data points corresponding to a given overall level of vector control intensity.
(TIFF)

S5 Fig **Impact of host mobility on the predicted vector control effectiveness within each of the following time windows:** (A) (0y, 5y], (B) (5y, 10y], (C) (10y, 15y], (D) (15y, 20y], (E) (20y, 25y], (F) (25y, 30y]. The parameter ω referred to the probability of biting a human living in a distant community per mosquito per bite. Each point represents a single simulation run, and the SHAP value quantified the change in the predicted vector control effectiveness due to the value of ω. Each curve was obtained by fitting a smoothing spline to the set of data points corresponding to a given overall level of vector control intensity.
(TIFF)

S6 Fig **Impact of antibody-dependent enhancement on the predicted vector control effectiveness within each of the following time windows**: (A) (0y, 5y], (B) (5y, 10y], (C) (10y, 15y], (D) (15y, 20y], (E) (20y, 25y], (F) (25y, 30y]. The parameter *ϕ*_*ADE*_ quantified both the susceptibility enhancement and transmissibility enhancement. Each point represents a single simulation run, and the SHAP value quantified the change in the predicted vector control effectiveness due to the value of *ϕ*_*ADE*_. Each curve was obtained by fitting a smoothing spline to the set of data points corresponding to a given overall level of vector control intensity.
(TIFF)

S7 Fig **Impact of the degree of antibody-dependent enhancement, average duration of cross-protection, and the degree of spatial variability in vector control intensity on the (A—C) critical time point, and (D—F) vector control effectiveness across the entire 30-year intervention period.** Each point represents a single simulation run, and the SHAP value quantified the change in the predicted value of the outcome variable due to the value taken by the predictor variable. Each curve was obtained by fitting a smoothing spline to the set of data points corresponding to a given overall level of vector control intensity. For (A—C), we excluded simulations where the overall level of vector control intensity was 0.8 or higher, due to the high probability that vector control remained effective throughout the 30-year intervention period.
(TIFF)

S8 Fig **Impact of cross-protection on the predicted vector control effectiveness within each of the following time windows**: (A) (0y, 5y], (B) (5y, 10y], (C) (10y, 15y], (D) (15y, 20y], (E) (20y, 25y], (F) (25y, 30y]. The parameter *l*_*C*_ referred to the average duration of cross-protection (measured in days). Each point represents a single simulation run, and the SHAP value quantified the change in the predicted vector control effectiveness due to the value of *l*_*C*_. Each curve was obtained by fitting a smoothing spline to the set of data points corresponding to a given overall level of vector control intensity.
(TIFF)

S9 Fig **Impact of spatial variability in vector control intensity on the predicted vector control effectiveness within each of the following time windows**: (A) (0y, 5y], (B) (5y, 10y], (C) (10y, 15y], (D) (15y, 20y], (E) (20y, 25y], (F) (25y, 30y]. The parameter *σ*_*RM*_ referred to the standard deviation of the community-level vector control intensity. Each point represents a single simulation run, and the SHAP value quantified the change in the predicted vector control effectiveness due to the value of *σ*_*RM*_. Each curve was obtained by fitting a smoothing spline to the set of data points corresponding to a given overall level of vector control intensity.
(TIFF)

S1 Text Supporting information. **Table A:** Summary of simulation output under different spatial distributions of breeding sites prior to the roll-out of the vector control intervention.
(PDF)

S1 Code Simulation code. (ZIP)
