# Supplementary figures and images for: Short-term and long-term epidemiological impacts of sustained vector control in various dengue endemic settings: A modelling study
Source: PLoS Comput Biol. 2022 Apr 1;18(4):e1009979. doi: 10.1371/journal.pcbi.1009979 (PMC8975162; doi:10.1371/journal.pcbi.1009979)

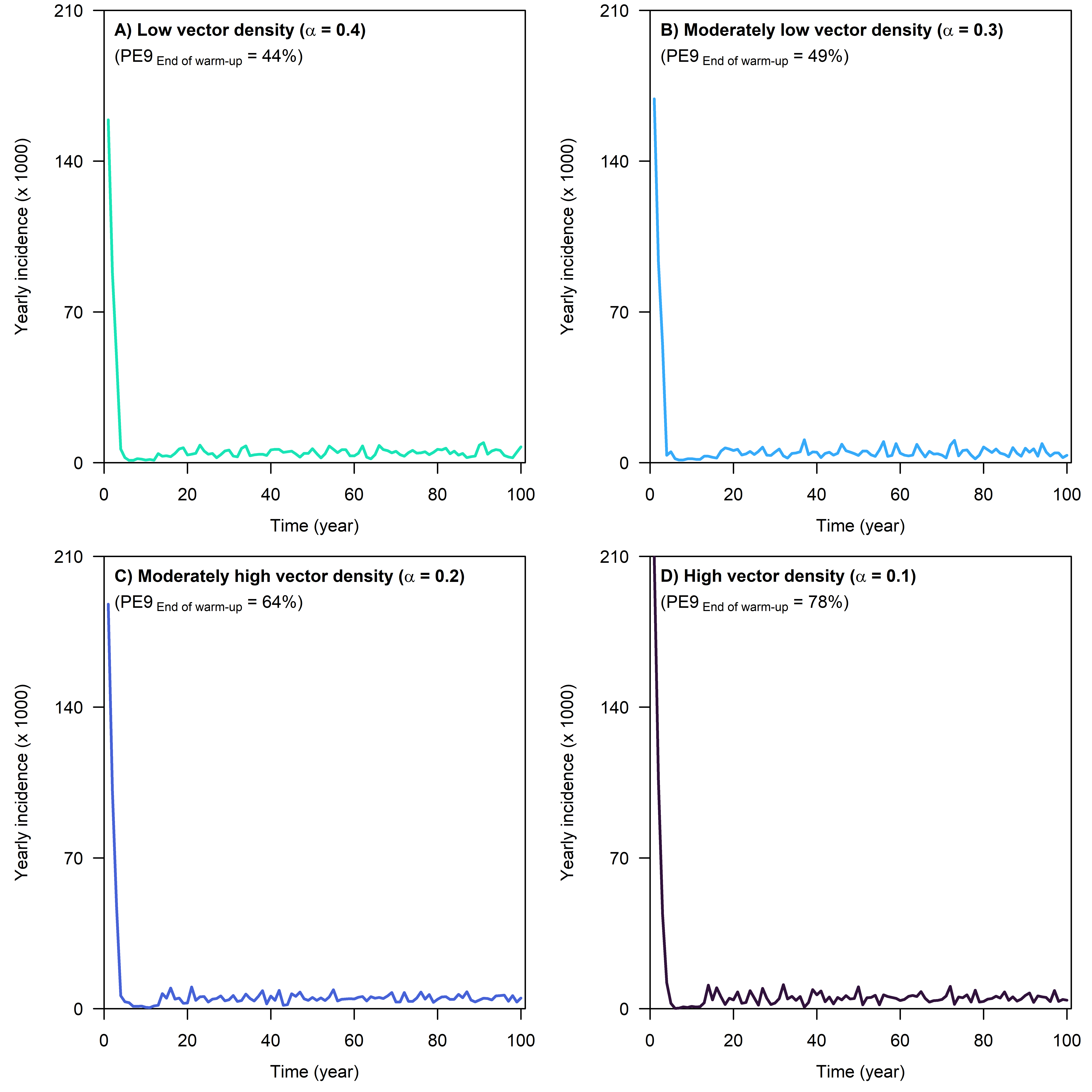

Supplement: S1 Fig — In all the figure panels, the yearly dengue incidence was found to converge within 20 years. (TIFF) [file pcbi.1009979.s001.tiff]

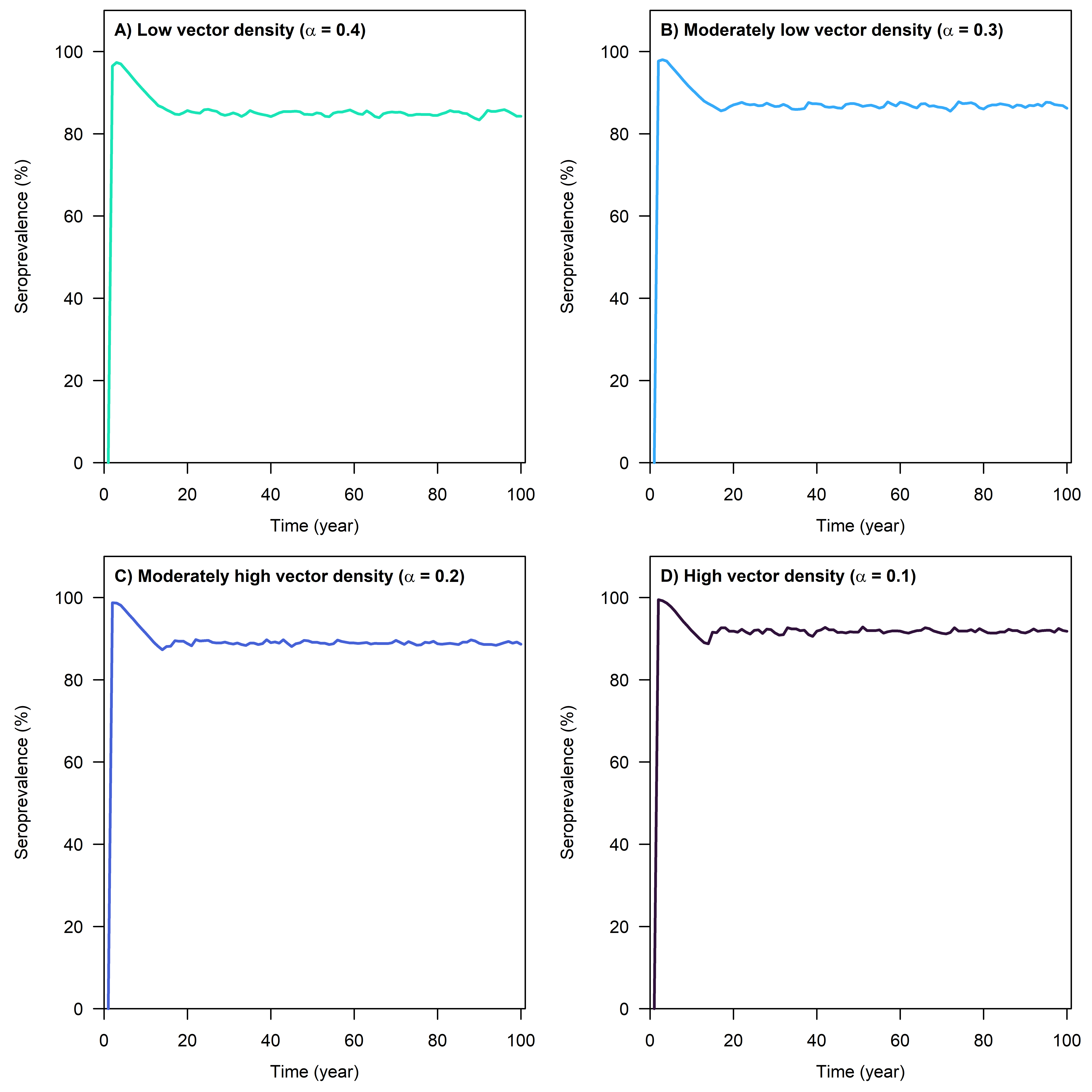

Supplement: S2 Fig — The four simulation runs were identical to those that generated the dengue incidence shown in S1 Fig, and in all the figure panels, dengue seroprevalence converged within 20 years. (TIFF) [file pcbi.1009979.s002.tiff]
