# Supplementary material for: Short-term and long-term epidemiological impacts of sustained vector control in various dengue endemic settings: A modelling study
Source: PLoS Comput Biol. 2022 Apr 1;18(4):e1009979. doi: 10.1371/journal.pcbi.1009979 (PMC8975162; doi:10.1371/journal.pcbi.1009979)
Supplement: S1 Text — Table A: Summary of simulation output under different spatial distributions of breeding sites prior to the roll-out of the vector control intervention. (PDF) [file pcbi.1009979.s010.pdf]

Short-term and long-term epidemiological impacts of sustained vector control in various dengue endemic settings: A modelling study

Haoyang Sun[1, *], Joel Koo[1], Borame L Dickens[1], Hannah E Clapham[1], Alex R Cook[1, *]

[1] Saw Swee Hock School of Public Health, National University of Singapore, Singapore

* haoyang.sun@nus.edu.sg (HS); * ephcar@nus.edu.sg (ARC)

Supporting Information

Non-uniform distribution of mosquito breeding sites

We created three scenarios as follows to test the robustness of our model results with respect to the spatial distribution of breeding sites prior to the roll-out of the intervention, with the total number of breeding sites held fixed. For each scenario, we first simulated dengue transmission during the 100-year warm-up period 200 times, each time using a unique random seed. For each random seed, dengue transmission during the 30-year intervention period was simulated four times, each time under a different overall level of vector control intensity: $p_{RM} = 0, 0.1, 0.5, 0.9$. To maximize the signal-to-noise ratio, the values of all the other model parameters were fixed across all the simulation runs: $\pi = 4 \times 10^{-5}, \omega = 0.005,\ \rho = 0,\ \sigma_{v_{h(2)}} = 0.5,\ \alpha = 0.25,\ \phi_{ADE} = 0.5,\ l_C = 180,\ \sigma_{RM} = 0$ (Here we used $\sigma_{RM} = 0$ to denote spatially uniform vector control intensity).

Scenario A

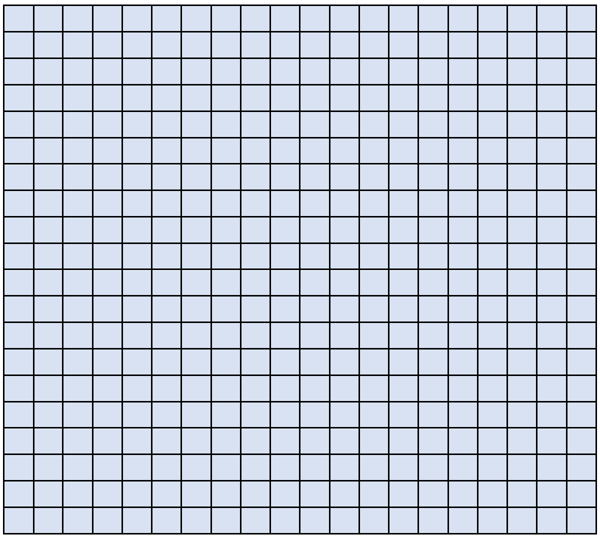

200 breeding sites per grid

Scenario B

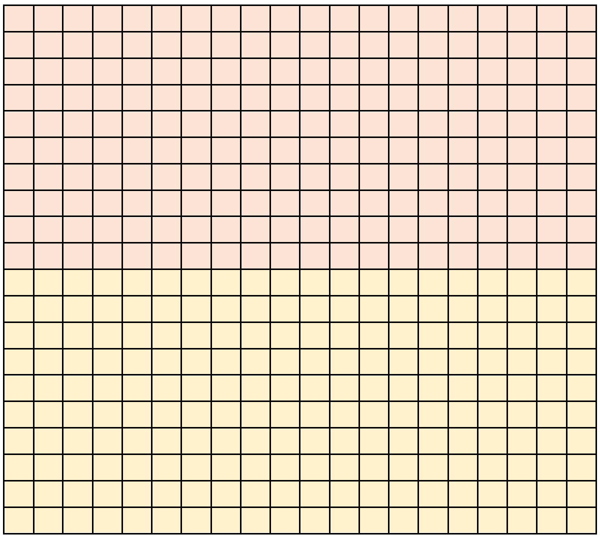

250 breeding sites per grid

150 breeding sites per grid

Scenario C

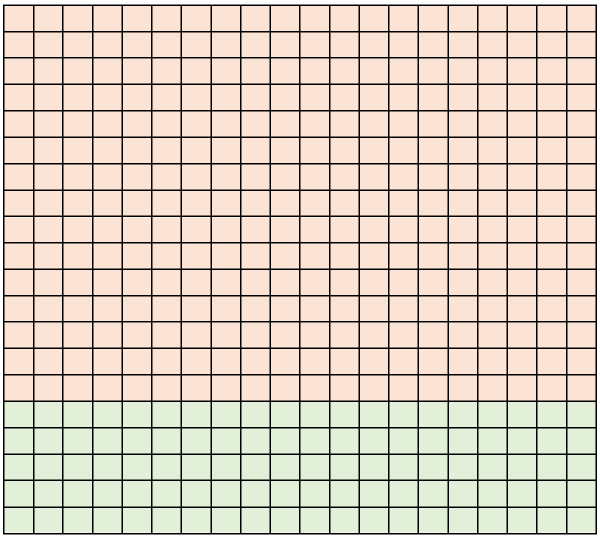

250 breeding sites per grid

50 breeding sites per grid

		Scenario A	Scenario B	Scenario C
Baseline PE9 (%)		52.9 (3.0)	51.9 (3.4)	48.8 (2.8)
Overall intervention effectiveness (%)	$p_{RM} = 0.1$	4.4 (2.7)	4.5 (2.3)	5.3 (2.7)
	$p_{RM} = 0.5$	33.3 (2.1)	33.9 (1.9)	37.0 (2.1)
	$p_{RM} = 0.9$	98.6 (0.1)	98.5 (0.2)	98.5 (0.2)
# of simulation runs where the 5-year intervention effectiveness remained positive throughout the 30-year intervention period	$p_{RM} = 0.1$	0	0	0
	$p_{RM} = 0.5$	0	0	4
	$p_{RM} = 0.9$	200	200	200
Critical time point (year)	$p_{RM} = 0.1$	2.8 (2.0)	3.1 (2.3)	2.9 (2.6)
	$p_{RM} = 0.5$	9.9 (1.6)	10.8 (2.0)	9.8 (2.2)
	$p_{RM} = 0.9$	-	-	-

Table A: Summary of simulation output under different spatial distributions of breeding sites prior to the roll-out of the vector control intervention. For the baseline PE9, overall intervention effectiveness and critical time point, results were summarized using the median value (interquartile range) in columns 'Scenario A', 'Scenario B', and 'Scenario C'.
